# Supplementary material for: IGF1-mediated human embryonic stem cell self-renewal recapitulates the embryonic niche
Source: Nat Commun. 2020 Feb 7;11:764. doi: 10.1038/s41467-020-14629-x (PMC7005693; doi:10.1038/s41467-020-14629-x)
Supplement: Supplementary file 1 — Supplementary Information_new [file 41467_2020_14629_MOESM1_ESM.pdf]

Supplementary Information

IGF1-mediated human embryonic stem cell self-renewal recapitulates the embryonic niche

Wamaitha et al.

Supplementary Figure 1

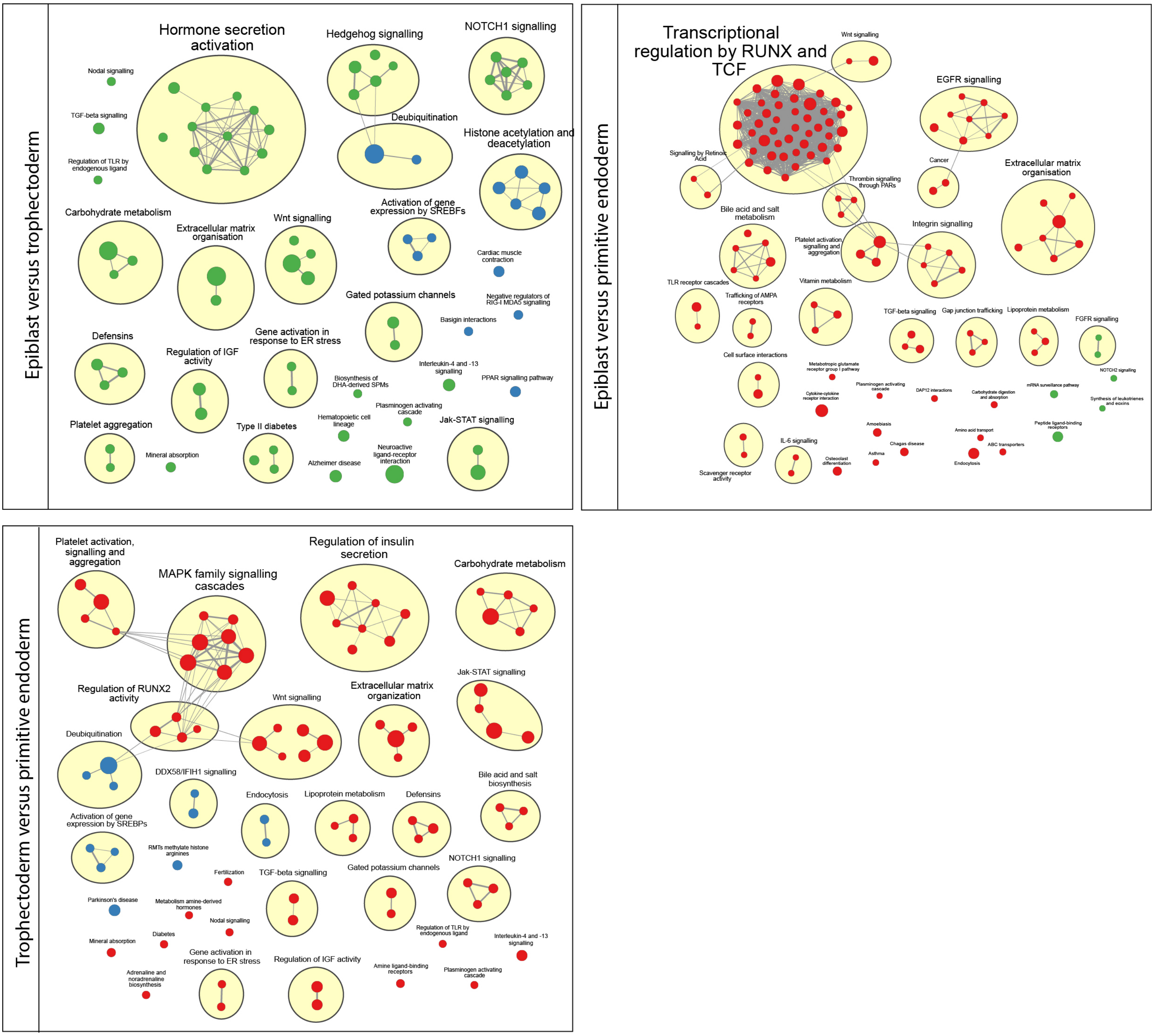

Supplementary Figure 1: EnrichmentMap constructed in Cytoscape comparing human blastocyst lineages following gene set enrichment analysis (P-value <0.01). Gene sets enriched in the Epi, PE or TE are coloured green, red or blue respectively.

Supplementary Figure 2

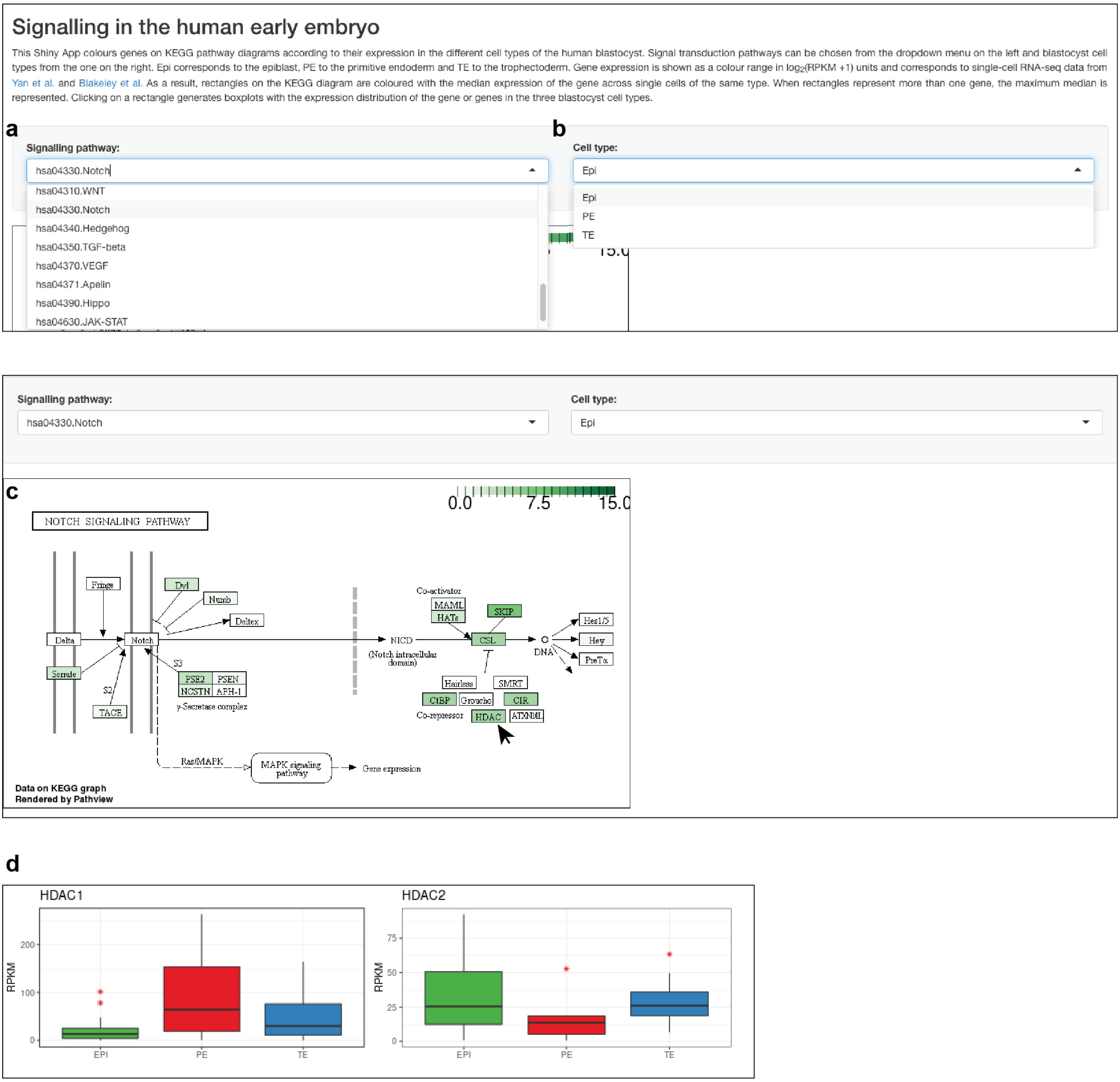

Supplementary Figure 2: An example of the website developed to search gene expression in the human blastocyst in the context of KEGG signal transduction pathways. Overview of the web tool user interface. (**a**) The 'Signalling pathway' dropdown menu on the left lists all the signal transduction pathways from the KEGG database. (**b**) The 'Cell type' menu on the right lists the three different cell types of the human early blastocyst (epiblast or Epi, primitive endoderm or PE, and trophectoderm or TE). (**c**) In this example, the NOTCH signalling pathway in the EPI lineage was selected. The graphic shows the level of expression of the NOTCH pathway members in the given lineage (EPI), with a colour-coded heat map in the top right. (**d**) Clicking on a specific pathway member (in this case *HDAC*) generates a boxplot showing the expression of the selected gene or genes (if the pathway member represents a gene category) in the three embryonic cell types. Boxes symbolise first and third quartiles, horizontal lines the median, whiskers extend to 1.5 times the interquartile range and the dots represent outliers. Web page can be accessed at: https://shiny.crick.ac.uk/embryo_signalling/

Supplementary Figure 3

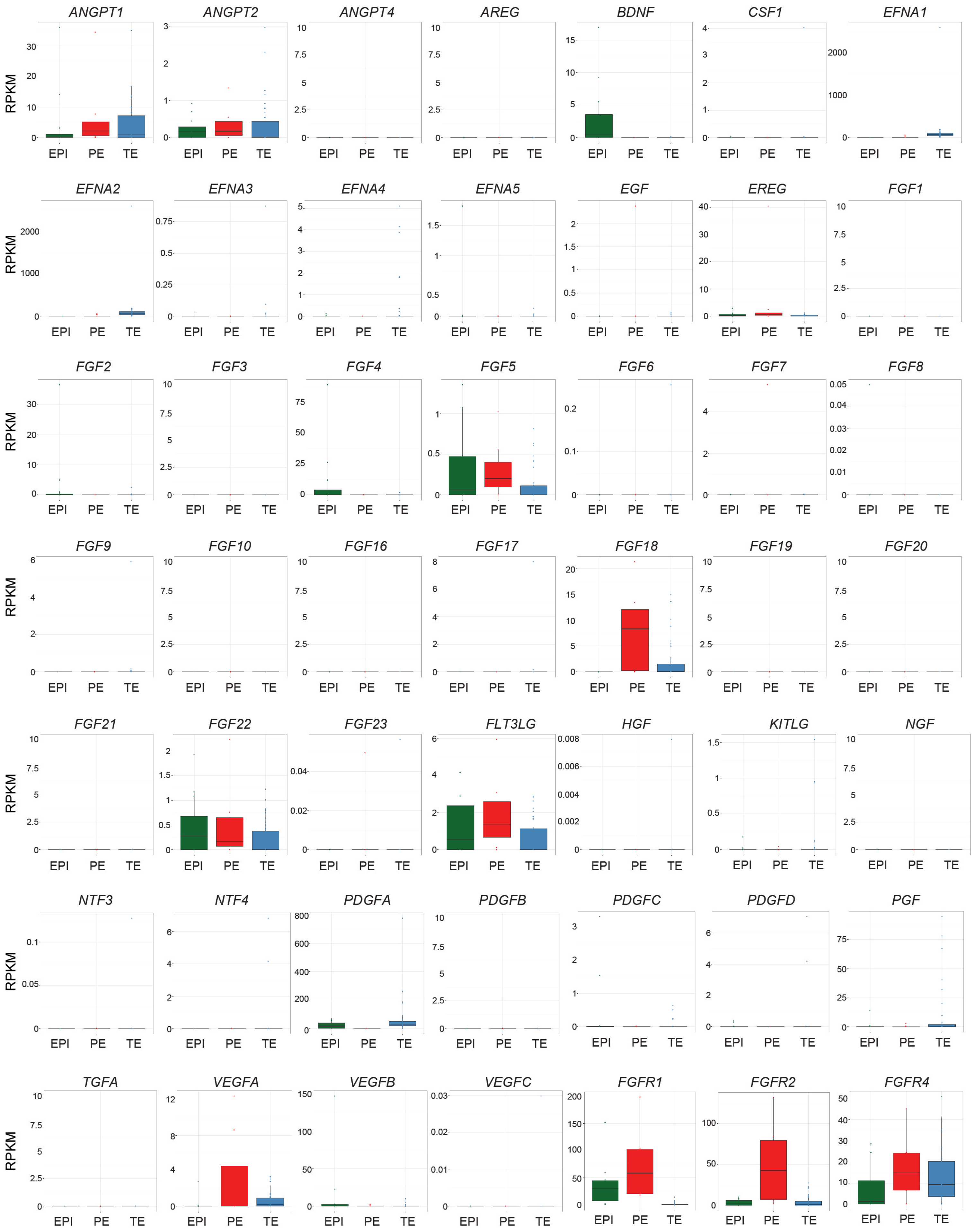

Supplementary Figure 3: Boxplot of RPKM values for MAPK ligands and selected FGF receptors in human preimplantation embryos. Trophectoderm (TE, blue); epiblast (EPI, green) and primitive endoderm (PE, red) expression is shown. Boxes symbolise first and third quartiles, horizontal lines the median, whiskers extend to 1.5 times the interquartile range and dots represent outliers.

Supplementary Figure 4

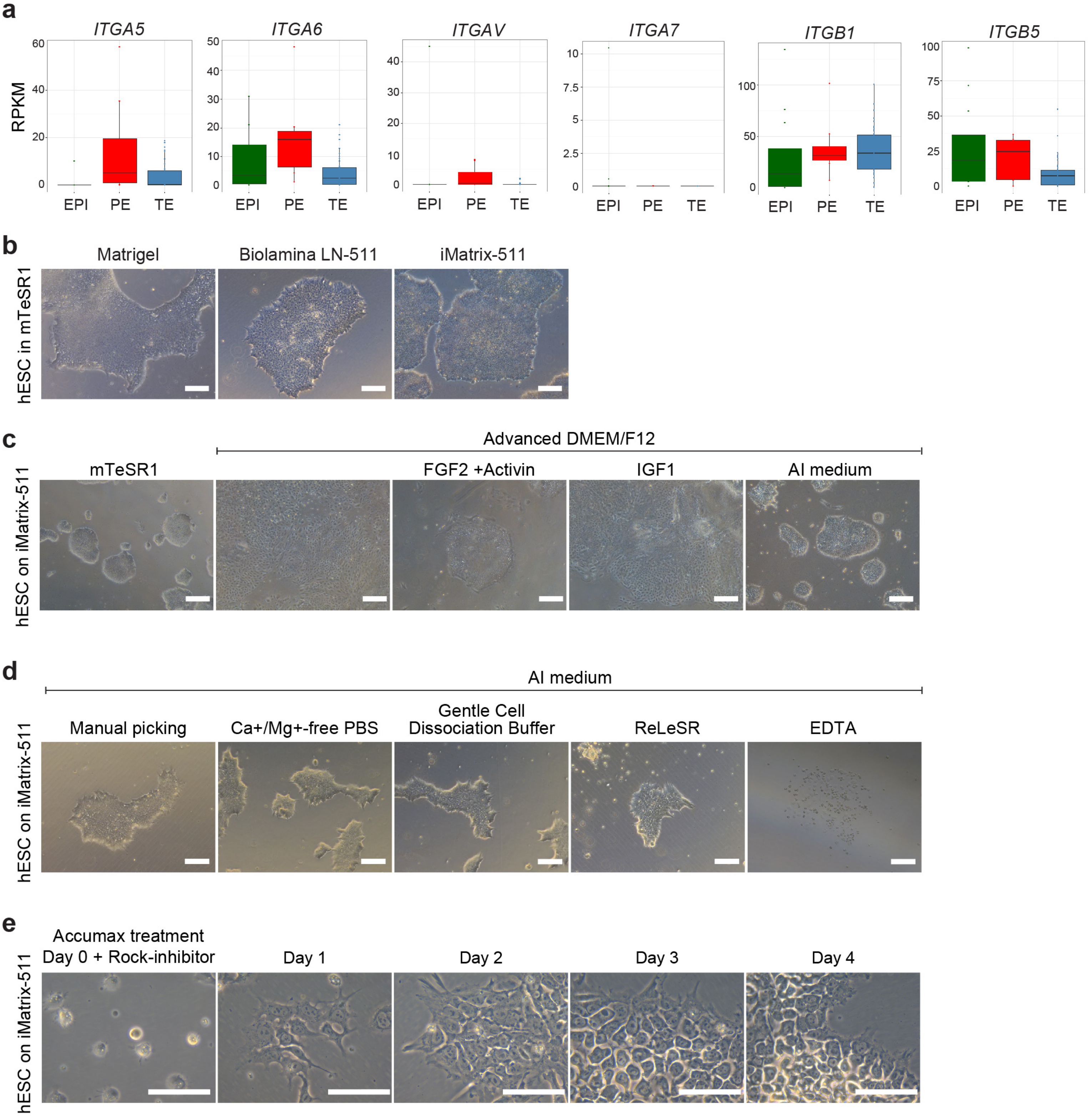

Supplementary Figure 4: Establishing Activin and IGF1 (AI) hESC culture medium. (**a**) Boxplots of reads per kilobase of million mapped reads (RPKM) values for integrin subunits (*ITGA5, ITGA6, ITGA7, ITGAV ITGB1, ITGB5*) in human blastocyst lineages (EPI green, PE red, TE blue) as determined by single cell RNA-seq[1]. Boxes symbolize first and third quartiles, horizontal lines the median, whiskers extend to 1.5 times the interquartile range and dots represent outliers. (**b**) Representative phase-contrast images of mTeSR1-cultured hESCs on Matrigel, Biolamina LN-511 and iMatrix-511 (Takara). Scale bar: 300 µm. *n* = 2 biological replicates. (**c**) Representative phase-contrast images of hESCs grown for two passages on Laminin-511 (iMatrix-511) in control mTeSR1 medium, basal medium (Advanced-DMEM/F12 plus 2 mM glutamine supplement) alone, or basal medium supplemented with 10 ng/ml Activin and 12 ng/ml FGF2; 17 nM IGF1; or 10 ng/ml Activin and 17 nM IGF1 (AI medium). *n* = 2 biological and 2 technical replicates. Scale bar: 300 µm. (**d**) Representative images of AI-adapted hESCs, 2 days after passage either manually, or with various dissociation reagents - Ca^+/Mg^+-free PBS, Gentle Cell Dissociation Reagent, ReLeSR or 0.5 mM EDTA. *n* = 2 or 3 technical replicates. Scale bar: 300 µm. (**e**) Representative images of hESCs dissociated with Accumax to single-cells in the presence of a Rho-associated kinase (ROCK) inhibitor and their subsequent growth. *n* = 3 biological and 3 technical replicates. Scale bar: 100 µm.

Supplementary Figure 5

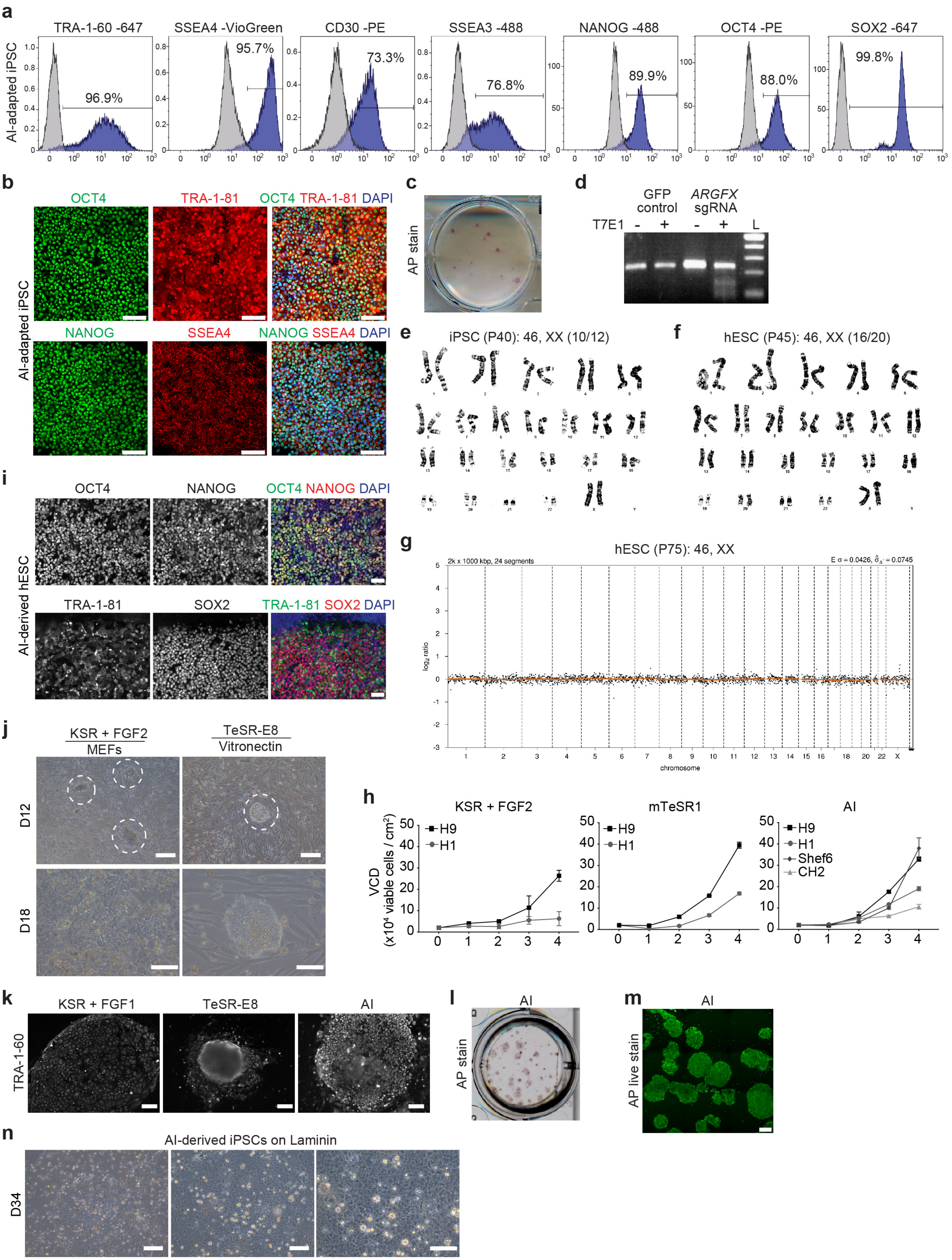

Supplementary Figure 5: Characterizing AI-adapted and *de novo* AI-derived hESCs and iPSCs. (**a**) Representative flow cytometry for AI-adapted RCiB10 iPSCs quantifying proportional expression of the indicated proteins (blue). Isotype control in grey. $n = 3$ technical replicates. (**b**) Representative immunofluorescence analysis of AI-adapted RCiB10 iPSCs for the indicated proteins; DAPI (blue) nuclear staining. $n = 3$ biological replicates. Scale bar: 50 μm. (**c**) Alkaline phosphatase staining following CRISPR/Cas9 targeting of *ARGFX* and clonal expansion of AI-cultured hESCs. (**d**) T7 endonuclease mismatch detection assay following nucleofection of a GFP control plasmid or a plasmid containing the gene encoding a Cas9 enzyme and a gRNA targeting *ARGFX*. T7 endonuclease I enzyme treated (+) or untreated (-) DNA is noted. Genomic ladder (L). (**e, f**) Representative G-banding patterns of AI-adapted iPSCs and hESCs. $n = 3$ biological replicates. (**g**) Representative karyotype analysis following whole-genome sequencing of AI-adapted hESCs. $n = 2$ biological and 2 technical replicates. (**h**) Viability assay for hESCs in AI medium ($n = 4$ biological and 3 technical replicates) compared to KSR+FGF2 and mTeSR1 media ($n = 2$ biological and 3 technical replicates). (**i**) Representative immunofluorescence analysis of AI-derived hESCs for the indicated proteins; DAPI nuclear stain (blue). $n = 3$ biological replicates. Scale bar: 50 μm. (**j**) Representative images of iPSC-like colonies (circled) derived from BJ fibroblasts with Sendai viruses driving the exogenous expression of OCT4, SOX2, KLF4 and cMYC (OSKM). Shown in KSR+FGF2 and TeSR-E8 media 12 and 18 days post-induction. $n = 2$ technical replicates. Scale bar: 300 μm top panels, 100 μm bottom panels. (**k**) TRA-1-60 staining of iPSC colonies in KSR+FGF2, TeSR-E8 or AI media 18 days post-induction. Scale bars: 100 μm. (**l**) Alkaline phosphatase staining of AI-derived iPSCs 18 days post-transduction. (**m**) Representative images of iPSC-like colonies derived following transfection of MRC5 fibroblasts with non-modified OSKM RNAs. Scale bar: 200 μm. (**n**) Phase contrast representative images of AI-derived iPSC lines maintained on laminin, 33 days post OSKM RNA transfection. Scale bar: left to right: 300 μm, 150 μm, 100 μm. Source data are provided as a Source Data file.

Supplementary Figure 6

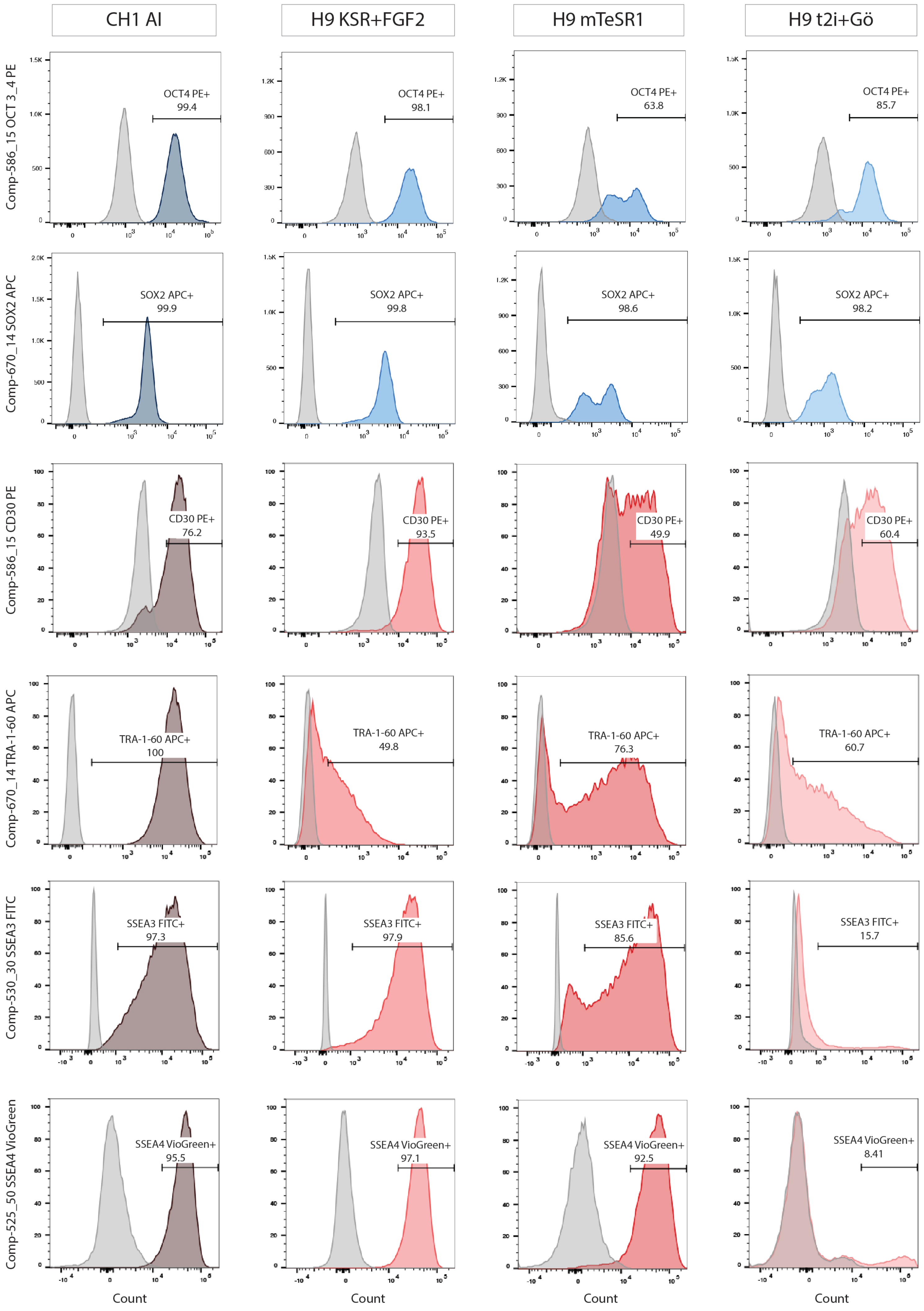

Supplementary Figure 6: Flow cytometry analysis of hESCs cultured in mTeSR1, KSR+FGF, t2iL+Gö or AI medium. Representative flow cytometry for H9 hESCS in t2iL+Gö, mTeSR1 and KSR+FGF2 compared to AI-derived CH3 hESCs. The proportion of cells expressing OCT4 and SOX2 (blue), and CD30, TRA-1-60, SSEA4 and SSEA3 (red), was quantified. Isotype control shown in grey.

Supplementary Figure 7

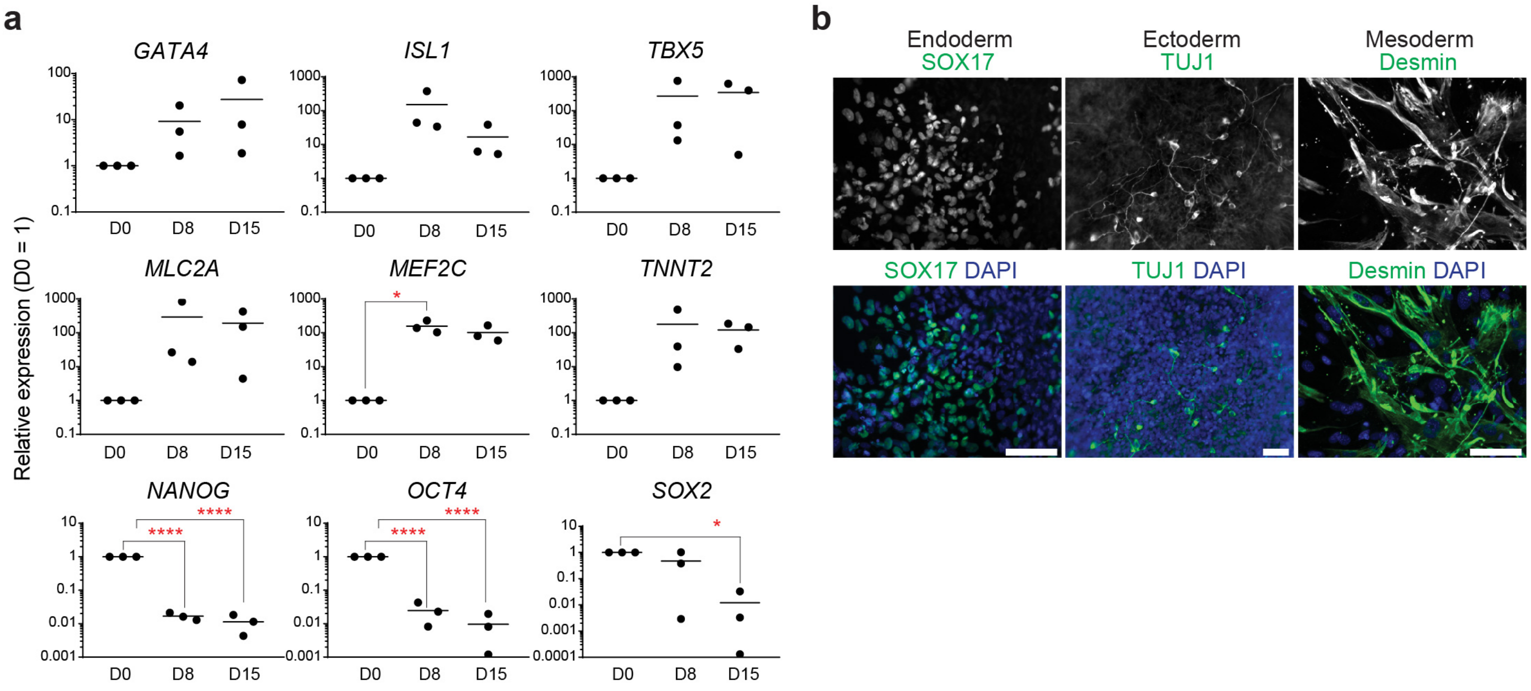

Supplementary Figure 7: Differentiation potential of cells cultured in AI medium. (**a**) qRT-PCR analysis following directed differentiation of AI-adapted hESCs using the STEMdiff cardiomyocyte differentiation kit (StemCell Technologies). Selected genes associated with hESCs (*NANOG*, *OCT4*, *SOX2*), foregut endoderm (*GATA4*, *ISL1*, *TBX5*) or cardiomyocytes (*MLC2A*, *MEF2C*, *TNNT2*). Relative expression as fold difference over undifferentiated hESCs (day 0 expression = 1) and normalized to *PBDG*. Data points and mean ± s.e.m. are shown: $n = 3$ biological and 2 technical replicates. One-way ANOVA; *$P < 0.05$; **$P < 0.01$; ***$P < 0.001$; ****$P < 0.0001$. (**b**) Representative immunofluorescence images following spontaneous differentiation of AI-derived hESCs into the three germ layers: SOX17 (endoderm), TUJ1 (ectoderm) and DESMIN (mesoderm) in green, DAPI nuclear stain in blue. $n = 3$ biological and 2 technical replicates. Scale bars: 100 μm. Source data are provided as a Source Data file.

Supplementary Figure 8

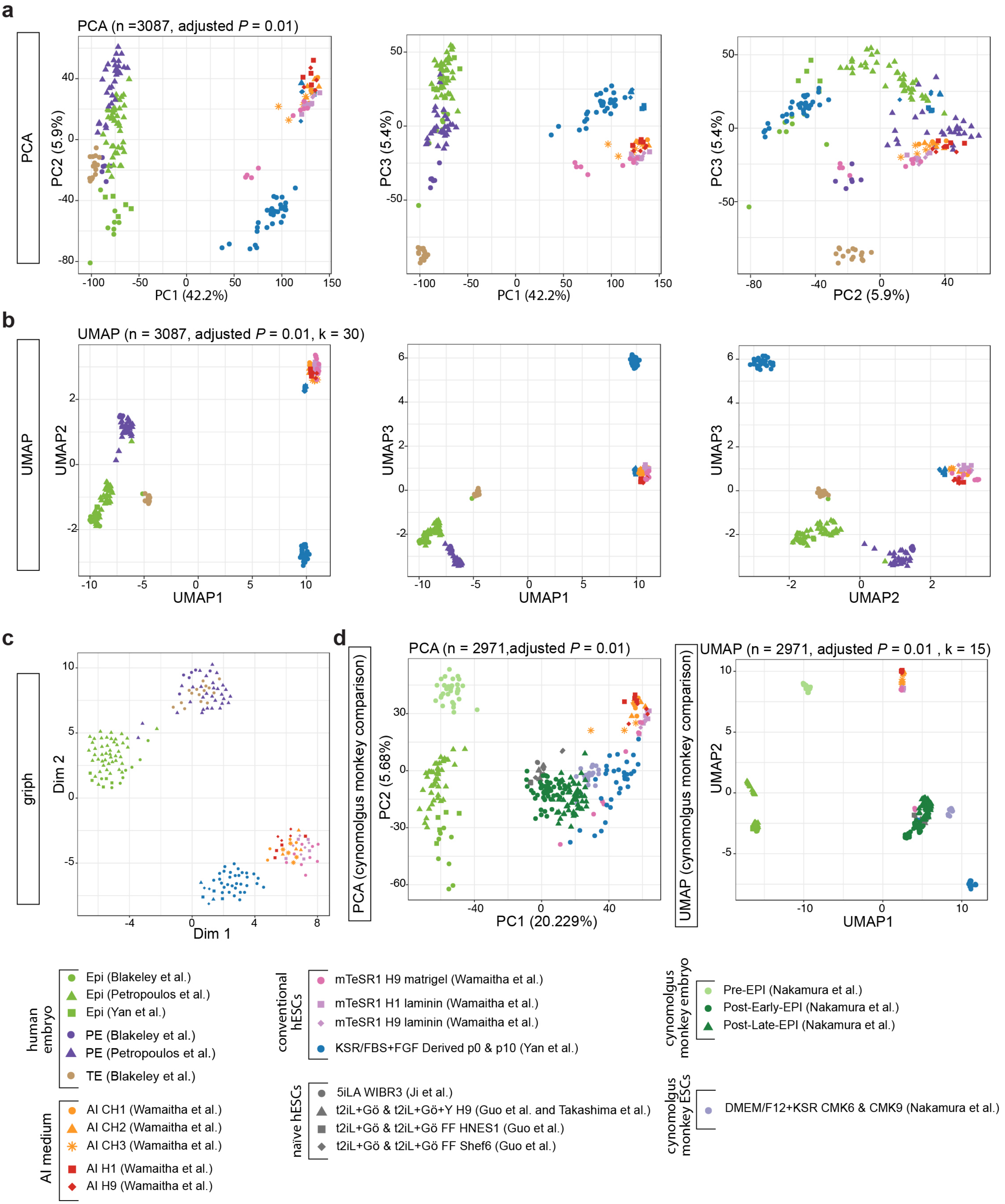

Supplementary Figure 8: Transcriptome analysis of hESCs and early human embryo cells. Analysis of single cell RNA-seq data from AI- or mTeSR1-cultured hESCs or human embryo EPI, PE or TE cells[1,2,3] (n = 3087 most variable genes, adjusted P ≤ 0.01, lowest Kullback-Leibler divergence from 100 runs). A color-coded sample key is provided. (**a**) Principal component analysis (PCA) plot showing the relationships between samples as captured by the first three principal components after removal of batch effects. (**b**) UMAP non-linear dimensionality reduction analysis of the same sample set. The initial number of neighbours was set to k = 30. (**c**) Clusters detected by griph when applied to RNA-seq data from the human embryo, conventional hESCs, and AI-cultured hESCs. (**d**) PCA and UMAP plots of single cell RNA-seq data from the human embryo and hESCs as above, as well as from hESCs in naïve media[4,5,6,7], and pre-implantation EPI and early or late postimplantation EPI cells of the cynomolgus monkey embryo[8] (n = 2971 most variable genes, adjusted P ≤ 0.01).

Supplementary Figure 9

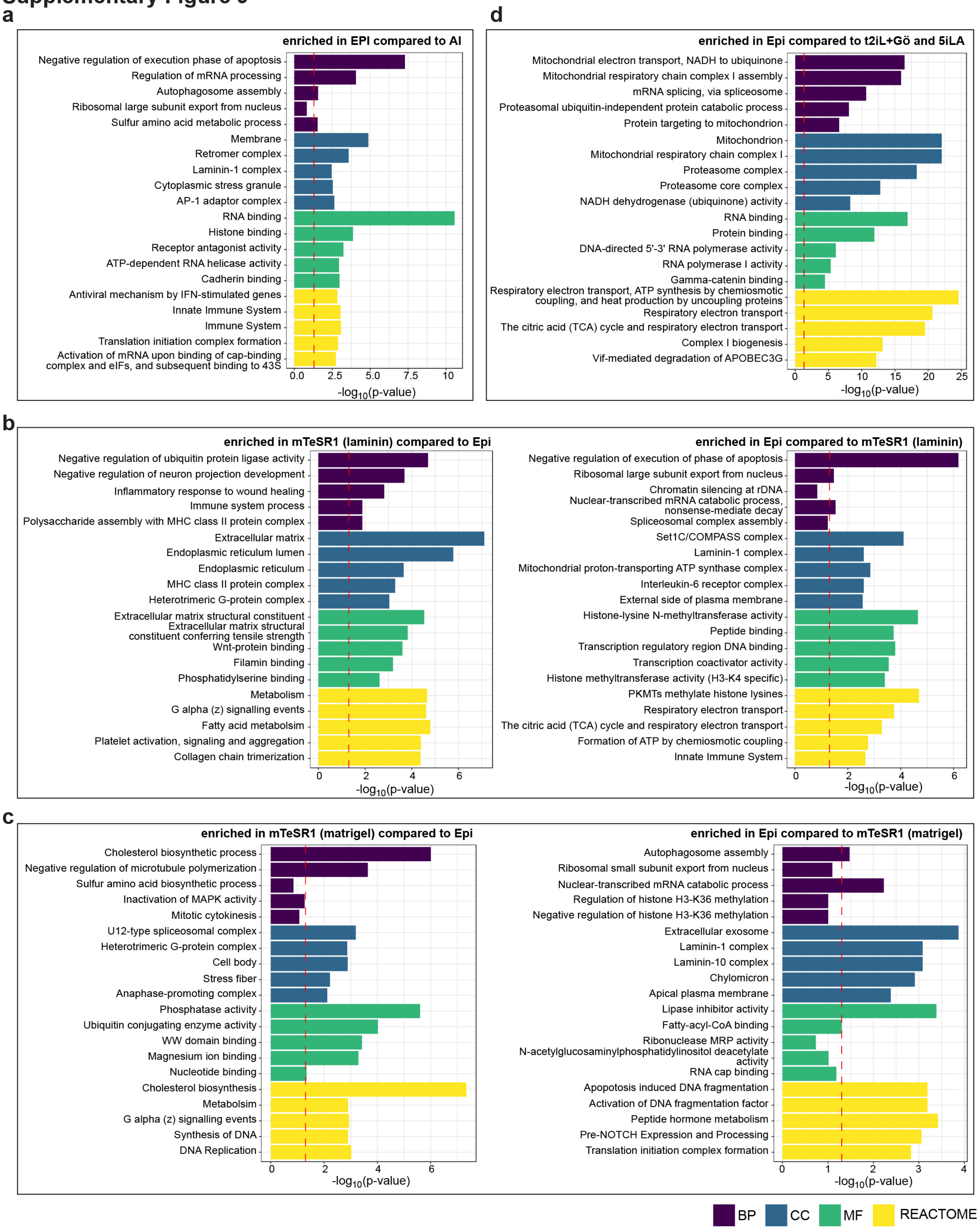

Supplementary Figure 9: Functional enrichment analysis of differentially expressed genes. The most enriched REACTOME pathways or GO terms associated with differentially expressed genes ($\log_2$ fold change ≥ 1, adjusted $P \leq 0.001$) are shown based on Benjamini-Hochberg corrected p-values (BP: biological process, CC: cellular component, MF: molecular function). Red dashed lines correspond to the significance level $\alpha = 0.05$. Comparison of human EPI cells to hESCs cultured in (**a**) AI medium; (**b**) t2iL+Gö and 5iLA media; mTeSR1 medium (**c**) laminin-511 or (**d**) mTeSR1 medium on Matrigel.

Supplementary Figure 10

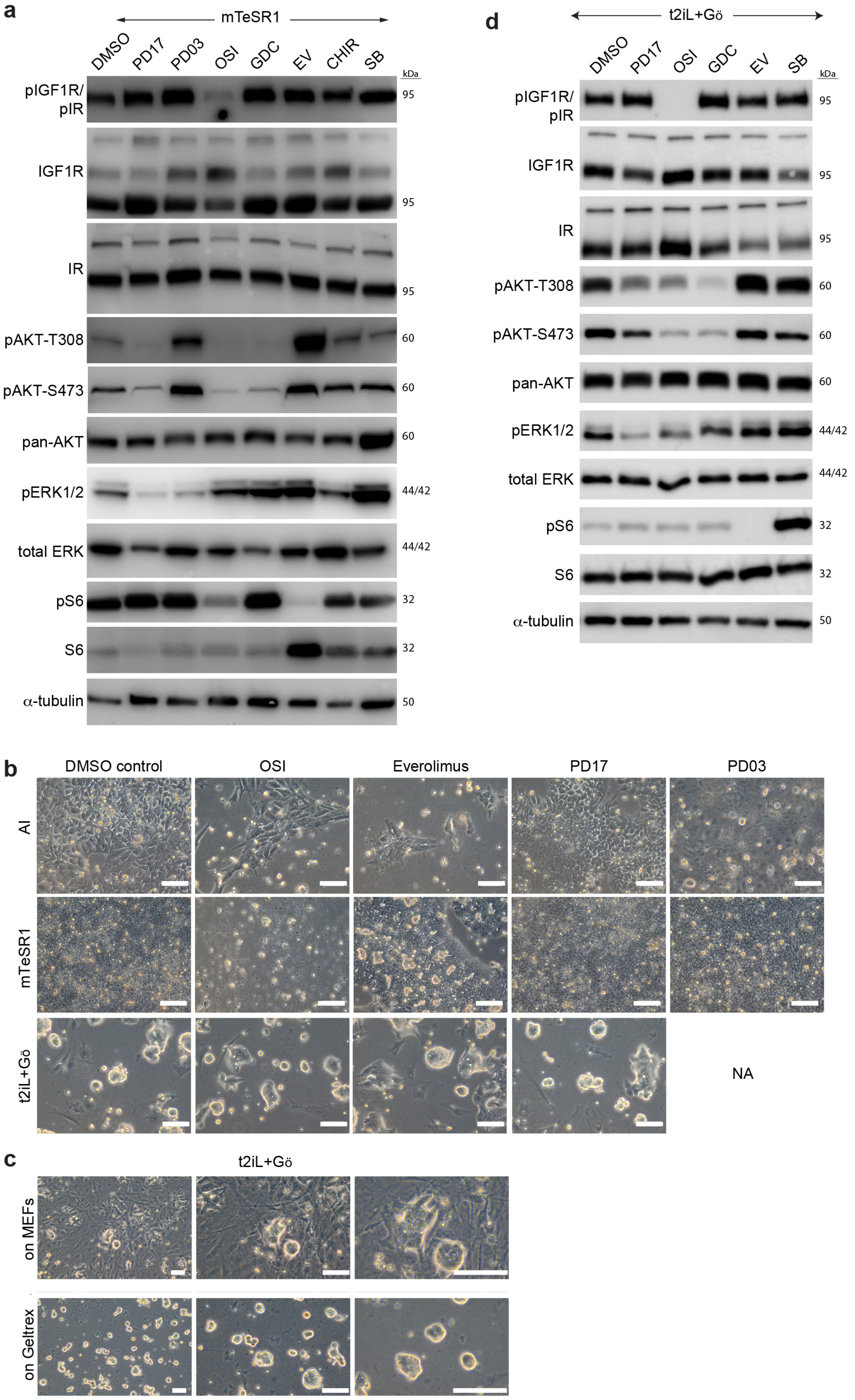

Supplementary Figure 10: Evaluating signalling pathway dynamics. (**a**) Representative western blots for proteins related to PI3K/AKT/mTOR and MAPK/ERK signalling in hESCs cultured in mTeSR1 medium supplemented with DMSO, PD17, PD03, OSI, GDC, EV, CHIR or SB for 24 hours. n = 2 biological and n = 3 technical replicates. (**b**) Representative phase contrast images of hESCs cultured in AI, mTeSR1 or t2iL+Gö medium with DMSO or with inhibitors for 3 days. Scale bars: 100 μm. n = 2 biological and n = 3 technical replicates. (**c**) Representative phase contrast images of hESCs cultured in t2iL+Gö on MEFs or on Geltrex substrate. Scale bars: 100 μm. (**d**) Representative western blots for proteins related to PI3K and MAPK/ERK signalling in hESCs cultured on Geltrex in t2iL+Gö medium supplemented with DMSO, PD17, OSI, GDC, EV or SB for 24 hours. n = 2 biological and n = 3 technical replicates. Full blots are provided in Supplementary Figure 11.

Supplementary Figure 11

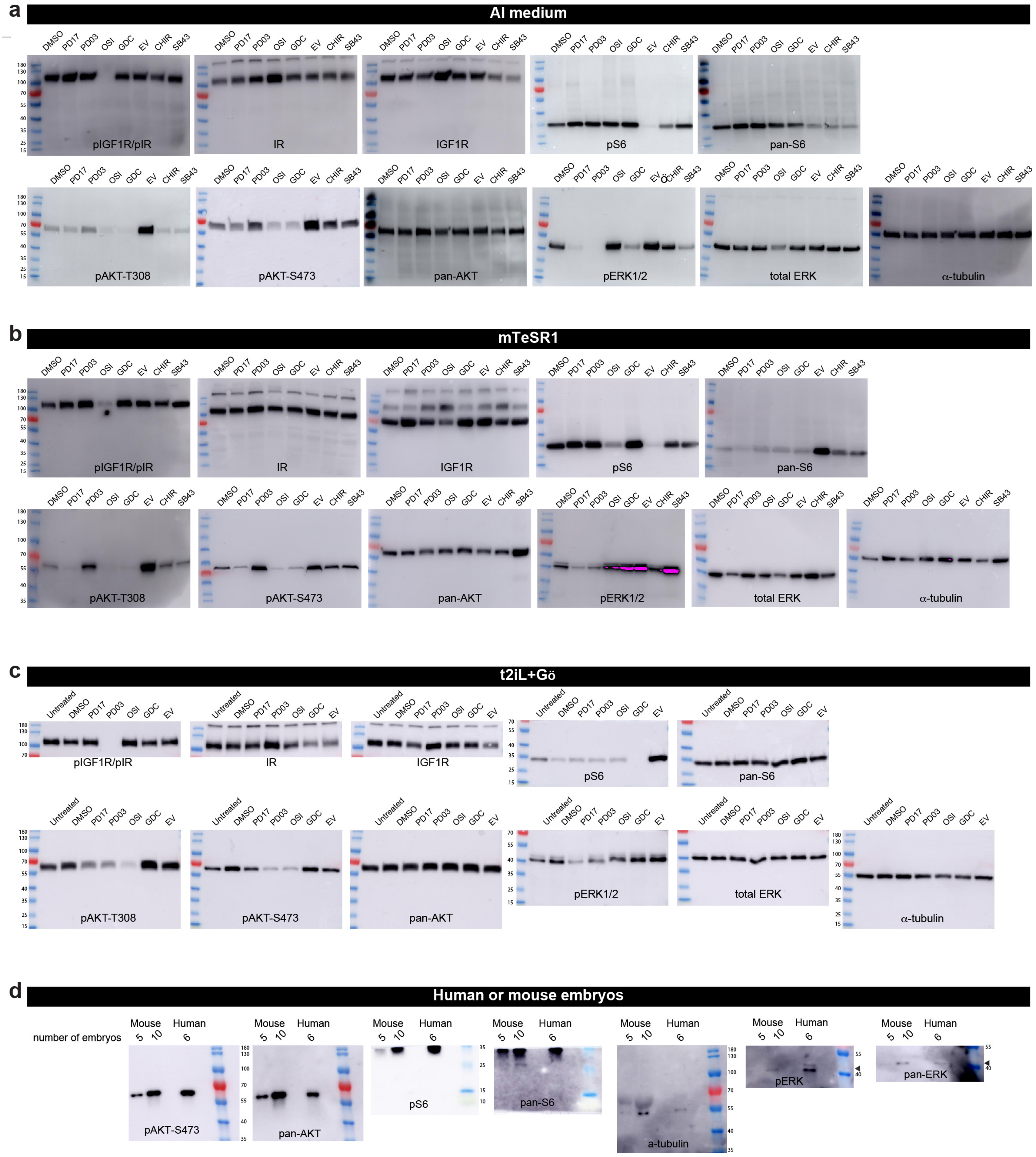

Supplementary Figure 11: Full-length panels of western blots. The western blots shown were used in Figures 3 and 4, and Supplementary Figure 10 for hESCs cultured in (a) AI, (b) mTeSR1 or (c) t2iL+Gö media, and (d) human or mouse embryos.

Supplementary Figure 12

a

Intracellular proteins

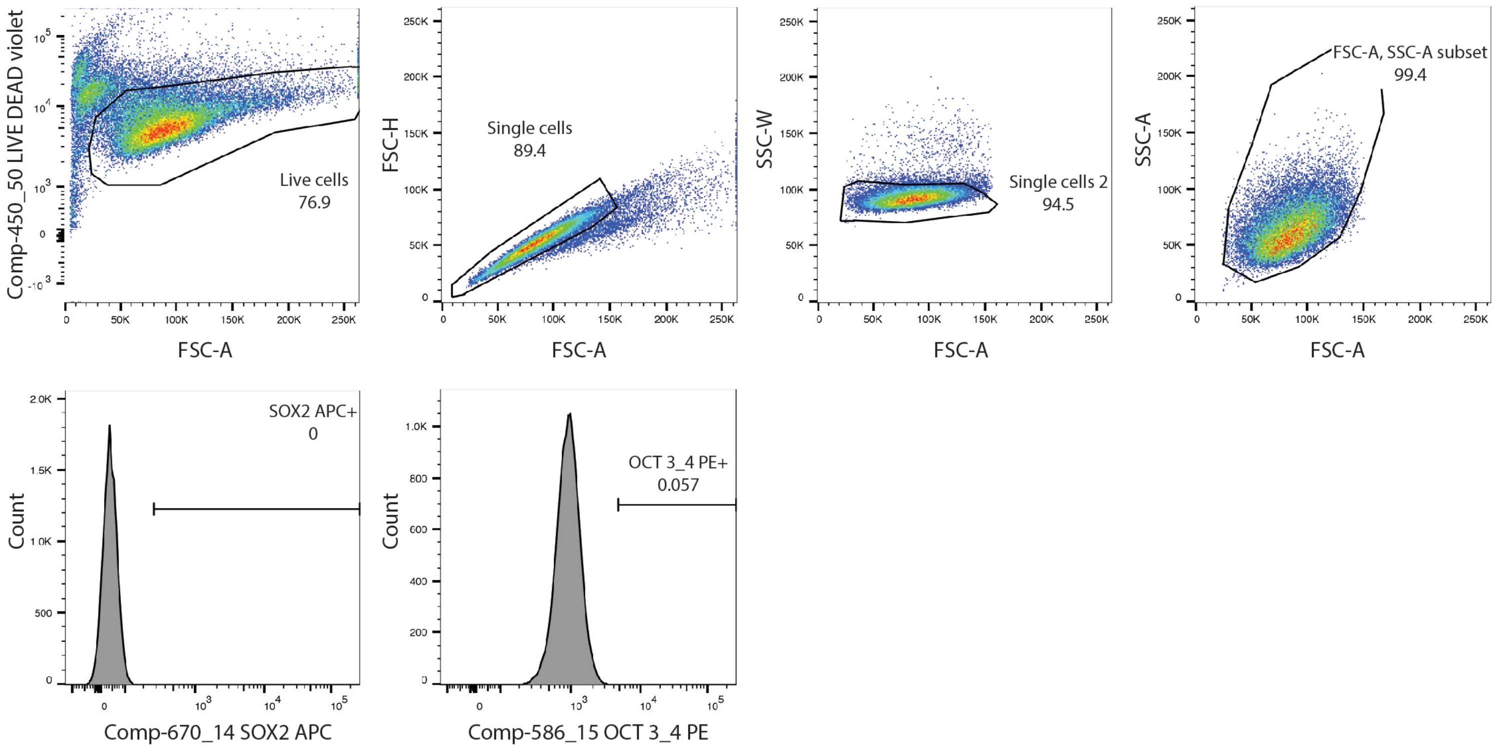

b

Surface marker proteins

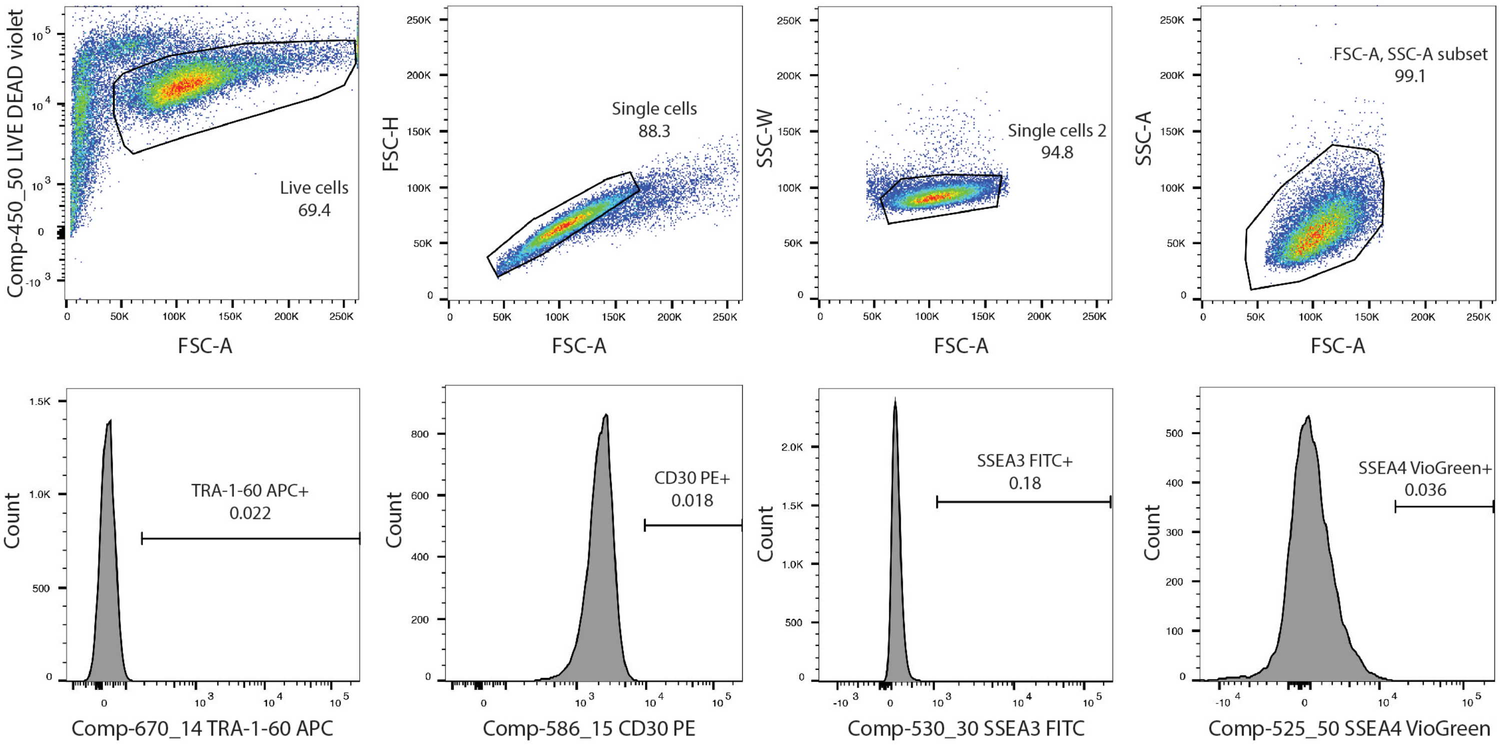

Supplementary Figure 12: Gating strategy for flow cytometry analysis of hESCs and iPSCs cultured in AI medium. Representative gating strategy and results for flow cytometry analysis of (**a**) intracellular and (**b**) cell surface marker proteins. Live cells were selected using a LIVE/DEAD® Fixable Violet Dead Cell Stain Kit. Cells were gated based on forward and side scatter plot of total events acquired, with doublets excluded. This live single cell population was then used for further expression analysis of surface (TRA1-60, CD30, SSEA-3, SSEA-4) and intracellular (NANOG, OCT-3/4, SOX2) markers. n = 3 technical replicates and 2 biological replicates.

Supplementary Tables

Supplementary Table 1: Primary antibodies used for IHC and Western blot

Primary Antibody	Supplier	Catalogue Number	Application	Species	Dilution
AFP	Dako	A0008	IHC	Rabbit	1:500
Alpha-Actin	Sigma	A7811	IHC	Mouse	1:400
Alpha-tubulin	Sigma	T9026	WB	Mouse	1:1000
Pan Akt	Cell Signaling	2920	WB	Mouse	1:1000
Phospho Akt S473	Cell Signaling	13038	WB	Rabbit	1:1000
Phospho Akt T308	Cell Signaling	4060	WB	Rabbit	1:1000
cTnT (cardiac troponin)	ThermoFisher	MA5-12960	IHC	Mouse	1:25
CK18	Abcam	ab668	IHC	Mouse	1:500
CXCR4	R&D	MAB173	IHC	Mouse	1:500
Desmin	Neomarkers	RB-9014-R7	IHC	Rabbit	1:50
FOXA2	Cell Signaling	3143	IHC	Rabbit	1:500
GATA6	Santa Cruz	SC-9055	IHC	Rabbit	1:500
KLF17	Atlas	HPA024629	IHC	Rabbit	1:250
IGF1R	Cell Signaling	3027	WB	Rabbit	1:1000
IR	Cell Signaling	3020	WB	Mouse	1:1000
Phospho IGF1R/IR	Cell Signaling	3024	WB	Rabbit	1:1000
Phospho p44/42 MAPK (Erk1/2)	Cell Signaling	9107	WB	Mouse	1:1000
Total p44/42 MAPK (Erk1/2)	Cell Signaling	4370	WB	Rabbit	1:1000
NANOG	Abcam	ab21624	IHC	Rabbit	1:500
NANOG	Cell Signaling	4903P	IHC	Rabbit	
NANOG	R&D	AF1997	IHC	Goat	1:500
NESTIN	Proteintech	66259	IHC	Mouse	1:500
NKX2.5	Santa Cruz	SC-8697	IHC	Goat	1:200
OCT4	Cell Signaling	2750S	IHC	Rabbit	
OCT4	Santa Cruz Biotech	SC-5279	IHC	Mouse	1:500
OTX2	R&D	AF1979	IHC	Goat	1:100
PAX6	Covance	PRB-278P	IHC	Rabbit	1:250
Pan S6	Cell Signaling	2317	WB	Mouse	1:1000
Phospho S6	Cell Signaling	2211	IHC/WB	Rabbit	1:1000 / 1:25
SOX17	R&D	AF1924	IHC	Goat	1:500
SSEA4	DSHB	MC-813-70	IHC	Mouse	1:100

SSEA4	Life Technologies	MA1-021	IHC	Mouse	
TRA-1-60	Millipore	MAB	IHC	Mouse IgM	1:100
TRA-1-81	Millipore	MAB4381	IHC	Mouse IgM	1:500
TRA-1-81	BD Biosciences	560072	IHC	Mouse IgM	
Tuj1	Sigma	T2200	IHC	Rabbit	1:500 / 1:250

Supplementary Table 2: Secondary antibodies used for IHC and Western blot

Secondary Antibody	Supplier	Catalogue No.	Application	Species	Dilution
Alexa Fluor anti-mouse IgG	Invitrogen	A21202 (488)<br>A21203 (594)<br>A31571 (647)	IHC	Donkey	1:300
Alexa Fluor anti-rabbit IgG	Invitrogen	A21206 (488)<br>A21207 (594)<br>A31573 (647)	IHC	Donkey	1:300
Alexa Fluor anti-goat IgG	Invitrogen	A11055 (488)<br>A11058 (594)<br>A21447 (647)	IHC	Donkey	1:300
Goat IgG (H+L), HRP conjugated	Santa Cruz	SC-2020	WB	Goat	1:20000
Mouse IgG (H+L), HRP conjugated	Cell Signaling	7076	WB	Mouse	1:20000
Rabbit IgG (H+L), HRP conjugated	Cell Signaling	7074	WB	Rabbit	1:20000

Supplementary Table 3: Conjugated antibodies used for flow cytometry

Primary Antibody (clone)	Supplier	Catalogue Number	Fluorochrome	Species
CD30 (Ber-H8)	BD Biosciences	550041	PE	Mouse
CD30, isotype control (MOPC-21)	BD Biosciences	555749	PE	Mouse
NANOG (N31-355)	BD Biosciences	560791	Alexa Fluor 488	Mouse
NANOG, isotype control (MOPC-21)	BD Biosciences	557702	Alexa Fluor 488	Mouse
OCT4 (O50-808)	BD Biosciences	561556	PE	Mouse
OCT4, isotype control (MOPC-21)	BD Biosciences	554680	PE	Mouse
SOX2 (14A6A34)	Biolegend	656108	Alexa Fluor 647	Mouse
SOX2, isotype control (MOPC-21)	Biolegend	400136	Alexa Fluor 647	Mouse
SSEA3 (MC-631)	Biolegend	330306	Alexa Fluor 488	Rat IgM
SSEA3, isotype control (RTK2118)	Biolegend	400811	Alexa Fluor 488	Rat IgM
SSEA4 (REA101)	Miltenyi Biotech	130-098-341	VioGreen	Human

SSEA4, isotype control (REA293)	Miltenyi Biotech	130-104-608	VioGreen	Human
TRA-1-60 (TRA-1-60-R)	Biolegend	330605	Alexa Fluor 647	Mouse
TRA-1-60, isotype control (MM-30)	Biolegend	401618	Alexa Fluor 647	Mouse

Supplementary Table 4: qRT-PCR primer pairs

**Name**	**Forward Primer**	**Reverse Primer**
GAPDH	GATGACATCAAGAAGGTGGTG	GTCTACATGGCAACTGTGAGG
GATA4	TCCCTCTTCCCTCCTCAAAT	TCAGCGTGTAAAGGCATCTG
ISL1	TGCTTTTCAGCAACTGGTCA	TGAATGTTCCTCATGCCTCA
MEF2C	ACGCGGATTATGGATGAACG	TGGCATACTGGAACAGCTTG
MESP1	ACCGTCCCCGCTCCTT	GTCCCTTGTCACTTGGGCT
MLC2A	AACTTGTCTGCCTGGGTCAG	AAGCCATCCTGAGTGCCTTC
NANOG	CATGAGTGTGGATCCAGCTTG	CCTGAATAAGCAGATCCATGG
OCT4	AGTGAGAGGCAACCTGGAGA	ACACTCGGACCACATCCTTC
OTX2	TGTGAAGACCTGTAGAACCTC	GGTTTGTAGGCCCCTCTAAG
PBDG1	ATTACCCCGGGAGACTGAAC	GGCTGTTGCTTGGACTTCTC
REX1	GGAATGTGGGAAAGCGTTCGT	CCGTGTGGATGCGCACGT
SOX2	TGGACAGTTACGCGCACAT	CGAGTAGGACATGCTGTAGGT
TBX5	GCTGGAAGGCGGATGTTT	GATCGTCGGCAGGTACAATG
TNNT2	TCCAGAAGACAGAGCGGAAA	CTTCATTCAGGTGGTCAATGG

Supplementary References

1. Blakeley, P. *et al.* Defining the three cell lineages of the human blastocyst by single-cell RNA-seq. *Development* **142**, 3613–3613 (2015).
2. Yan, L. *et al.* Single-cell RNA-Seq profiling of human preimplantation embryos and embryonic stem cells. *Nat. Struct. Mol. Biol.* **20**, 1131–1139 (2013).
3. Petropoulos, S. *et al.* Single-Cell RNA-Seq Reveals Lineage and X Chromosome Dynamics in Human Preimplantation Embryos. *Cell* **165**, 1012–1026 (2016).
4. Takashima, Y. *et al.* Resetting Transcription Factor Control Circuitry toward Ground-State Pluripotency in Human. *Cell* **158**, 1254–1269 (2014).
5. Ji, X. *et al.* 3D Chromosome Regulatory Landscape of Human Pluripotent Cells. *Cell Stem Cell* **18**, 262–75 (2016).
6. Guo, G. *et al.* Naive Pluripotent Stem Cells Derived Directly from Isolated Cells of the Human Inner Cell Mass. *Stem cell reports* **6**, 437–446 (2016).
7. Chan, Y.-S. *et al.* Induction of a Human Pluripotent State with Distinct Regulatory Circuitry that Resembles Preimplantation Epiblast. *Cell Stem Cell* **13**, 663–675 (2013).
8. Nakamura, T. *et al.* A developmental coordinate of pluripotency among mice, monkeys and humans. *Nature* **537**, 57–62 (2016).
