# Supplementary material for: IGF1-mediated human embryonic stem cell self-renewal recapitulates the embryonic niche
Source: Nat Commun. 2020 Feb 7;11:764. doi: 10.1038/s41467-020-14629-x (PMC7005693; doi:10.1038/s41467-020-14629-x)
Supplement: Supplementary file 10 — Reporting Summary [file 41467_2020_14629_MOESM10_ESM.pdf]

nature research

Corresponding author(s): Kathy Niakan

Last updated by author(s): January 3rd 2020

Reporting Summary

Nature Research wishes to improve the reproducibility of the work that we publish. This form provides structure for consistency and transparency in reporting. For further information on Nature Research policies, see Authors & Referees and the Editorial Policy Checklist .

Statistics

For all statistical analyses, confirm that the following items are present in the figure legend, table legend, main text, or Methods section.

n/a	Confirmed	
☐	☒	The exact sample size (*n*) for each experimental group/condition, given as a discrete number and unit of measurement
☐	☒	A statement on whether measurements were taken from distinct samples or whether the same sample was measured repeatedly
☐	☒	The statistical test(s) used AND whether they are one- or two-sided<br>*Only common tests should be described solely by name; describe more complex techniques in the Methods section.*
☐	☒	A description of all covariates tested
☐	☒	A description of any assumptions or corrections, such as tests of normality and adjustment for multiple comparisons
☐	☒	A full description of the statistical parameters including central tendency (e.g. means) or other basic estimates (e.g. regression coefficient) AND variation (e.g. standard deviation) or associated estimates of uncertainty (e.g. confidence intervals)
☐	☒	For null hypothesis testing, the test statistic (e.g. *F*, *t*, *r*) with confidence intervals, effect sizes, degrees of freedom and *P* value noted<br>*Give P values as exact values whenever suitable.*
☒	☐	For Bayesian analysis, information on the choice of priors and Markov chain Monte Carlo settings
☐	☒	For hierarchical and complex designs, identification of the appropriate level for tests and full reporting of outcomes
☒	☐	Estimates of effect sizes (e.g. Cohen's *d*, Pearson's *r*), indicating how they were calculated

Our web collection on statistics for biologists contains articles on many of the points above.

Software and code

Policy information about availability of computer code

Data collection	Details of the data collection and codes used can be found at the following site: https://github.com/galanisl/AI_hESCs.
Data analysis	A detailed analysis of the RNA-seq pipeline can be found at the following site: https://github.com/galanisl/AI_hESCs. A summary of the data analysis is included in the methods section.

For manuscripts utilizing custom algorithms or software that are central to the research but not yet described in published literature, software must be made available to editors/reviewers. We strongly encourage code deposition in a community repository (e.g. GitHub). See the Nature Research guidelines for submitting code & software for further information.

Data

Policy information about availability of data

All manuscripts must include a data availability statement. This statement should provide the following information, where applicable:

- Accession codes, unique identifiers, or web links for publicly available datasets
- A list of figures that have associated raw data
- A description of any restrictions on data availability

RNA-seq FastQ files have been deposited into the Gene Expression Omnibus repository (GSE126488). There are no restrictions on the data. This data was used to generate Figure 2, Supplementary Figures 8 and 9 and Supplementary Data 3, 4, 5 and 6.

Field-specific reporting

Please select the one below that is the best fit for your research. If you are not sure, read the appropriate sections before making your selection.

Life sciences study design

All studies must disclose on these points even when the disclosure is negative.

Sample size	We analysed at least 3 technical replicates of 2, 3 or 4 biological replicates per dataset shown and the number used for each figure is noted in the figure legend.
Data exclusions	No data was excluded from the analyses.
Replication	Attempts at replication were successful.
Randomization	Randomization was not performed in our studies.
Blinding	The computational analysis of cell lines and human embryos was performed blind to the identity of the samples.

Reporting for specific materials, systems and methods

We require information from authors about some types of materials, experimental systems and methods used in many studies. Here, indicate whether each material, system or method listed is relevant to your study. If you are not sure if a list item applies to your research, read the appropriate section before selecting a response.

Materials & experimental systems

n/a	Involved in the study	
☐	☒	Antibodies
☐	☒	Eukaryotic cell lines
☒	☐	Palaeontology
☒	☐	Animals and other organisms
☒	☐	Human research participants
☒	☐	Clinical data

Methods

n/a	Involved in the study	
☒	☐	ChIP-seq
☐	☒	Flow cytometry
☒	☐	MRI-based neuroimaging

Antibodies

Antibodies used	This information is included in Supplementary Tables 1, 2 and 3. Other details are in the methods section.
Validation	All of the antibodies used in this study have been previously published and their specificity has been validated either by us or others.

Eukaryotic cell lines

Policy information about cell lines

Cell line source(s)	The H1 and H9 hESC lines were obtained under licence and SLA agreement with WiCell. The Shef6 cell line was obtained from the UK Stem Cell Bank.
Authentication	All of the hESC lines used have been exhaustively tested include STA profiling, karyotpying, gene and protein expression and differentiation.
Mycoplasma contamination	The cell lines were routinely tested for mycoplasma and were found to be negative.
Commonly misidentified lines (See ICLAC register)	N/A

Flow Cytometry

Plots

Confirm that:

☒ The axis labels state the marker and fluorochrome used (e.g. CD4-FITC).

☒ The axis scales are clearly visible. Include numbers along axes only for bottom left plot of group (a 'group' is an analysis of identical markers).

☐ All plots are contour plots with outliers or pseudocolor plots.

☒ A numerical value for number of cells or percentage (with statistics) is provided.

Methodology

Sample preparation	Single cell suspensions were washed and stained in BD Pharmigen™ Stain Buffer (BD Biosciences) with conjugated primary antibodies (Supplementary Table 3). For intracellular proteins, cells were fixed using BD Cytofix™ fixation buffer (BD Biosciences) and incubated for 15 min at RT. Cells were then permeabilised with 0.1% Triton X-100 (Sigma) in BD Pharmigen™ Stain Buffer for 15 min at RT prior to staining. Isotype controls were performed for each antibody (Supplementary Table 3). Cells were stained with Live/Dead® discrimination dye (L23105, ThermoFischer Scientific) and phenotype analysis of the live single cell population fraction performed by flow cytometry. Isotype staining was considered as a negative control for each analysis and condition.
Instrument	Expression of surface- and intracellular-pluripotency related proteins was analysed using a MACSQuant® Analyzer10 flow cytometer (Miltenyi Biotech) or BD LSRFortessa™ flow cytometer (BD Biosciences).
Software	*Describe the software used to collect and analyze the flow cytometry data. For custom code that has been deposited into a community repository, provide accession details.*
Cell population abundance	*Describe the abundance of the relevant cell populations within post-sort fractions, providing details on the purity of the samples and how it was determined.*
Gating strategy	Cells were gated based on forward and side scatter plot of total events acquired (P1). Doublets were then excluded (P1/P2) and live cells (unstained) were selected using a LIVE/DEAD® Fixable Blue Dead Cell Stain Kit (P1/P2/P3). This live single cell population was then used for further expression analysis of surface and intracellular markers as indicated in the figure legend (P1/P2/P3/P4). Contour plots are shown in Supplementary Figure 12.

☒ Tick this box to confirm that a figure exemplifying the gating strategy is provided in the Supplementary Information.
