# Supplementary material for: IGF1-mediated human embryonic stem cell self-renewal recapitulates the embryonic niche
Source: Nat Commun. 2020 Feb 7;11:764. doi: 10.1038/s41467-020-14629-x (PMC7005693; doi:10.1038/s41467-020-14629-x)
Supplement: Supplementary file 11 — Description of Additional Supplementary Files [file 41467_2020_14629_MOESM11_ESM.pdf]

Title: Supplementary Data 1:
Description: Gene set enrichment analysis comparing human blastocyst EPI, TE and PE lineages. Gene sets were clustered with the AutoAnnotate tool in Cytoscape based on the number of genes they share. Raw (ES) and normalized enrichment (NES) scores, as well as nominal p-values are provided.

Title: Supplementary Data 2:
Description: Putative ligand receptor pairs from human preimplantation embryo RNA-seq data.

Title: Supplementary Data 3:
Description:DESeq2 comparison tables. Multiple differential gene expression comparisons between human blastocyst EPI, TE or PE lineages, AI-adapted (H1, H9) hESCs or AI-derived hESCs (CH1, CH2, CH3) on laminin, mTeSR1-cultured hESCs on Matrigel or laminin, and hESCs cultured in KSR+FGF or t2iL+Gö and 5iLA medium. Positive log2 fold change value = first name indicated in each tab and negative = second name indicated in each tab. The log2-fold changes (log2FoldChange), nominal (pvalue) and adjusted p-values (padj), the mean of the normalized counts of a gene across samples (baseMean), the standard error of the log2-fold changes (lfcSE) and the Wald statistic (stat) are reported.

Title: Supplementary Data 4:
Description: Gene set enrichment analysis comparing AI-cultured hESCs to the human EPI and hESCs cultured in alternative media. Raw (ES) and normalized enrichment (NES) scores, as well as nominal p-values are provided.

Title: Supplementary Data 5:
Description: Functional enrichment analysis comparing mTeSR1-cultured hESCs on Matrigel versus laminin-511. The most enriched REACTOME pathways or GO terms associated with differentially expressed genes (log2 fold change ≥ 1, adjusted P ≤ 0.001) are shown based on Benjamini-Hochberg corrected p-values (BP: biological process, CC: cellular component, MF: molecular function).

Title: Supplementary Data 6:
Description: RNA-seq normalized gene expression matrix data Supplementary Movies

Title: Supplementary Movie 1:
Description: Beating cardiomyocytes differentiated from hESCs previously cultured in AI medium.
